# Analysis of gastrointestinal virus infection in immunocompromised hosts by multiplex virus PCR assay

**DOI:** 10.3934/microbiol.2018.2.225

**Published:** 2018-03-19

**Authors:** Miho Sasaki, Norio Shimizu, Yuriko Zushi, Toshiharu Saito, Hiroko Tsunemine, Kiminari Itoh, Yumi Aoyama, Yuta Goto, Taiichi Kodaka, Goh Tsuji, Eri Senda, Takahiro Fujimori, Tomoo Itoh, Takayuki Takahashi

**Affiliations:** 1 Laboratory of Cell Therapy, Shinko Hospital, Kobe, Japan; 2 Departments of Virology, Medical Research Institute, Tokyo Medical and Dental University, Tokyo, Japan; 3 Laboratory of Hematology, Shinko Hospital, Kobe, Japan; 4 Laboratory of Rheumatology, Shinko Hospital, Kobe, Japan; 5 Laboratory of Gastroenterology and Hepatology, Shinko Hospital, Kobe, Japan; 6 Laboratory of Pathology, Shinko Hospital, Kobe, Japan; 7 Department of Diagnostic Pathology, Kobe University Graduate School of Medicine, Kobe, Japan

**Keywords:** multiplex virus PCR assay, gastrointestinal mucosa, CMV colitis, herpes family virus, viral gastroenteritis

## Abstract

Regarding viral infection of intestinal mucosa, there have been only a few studies on limited diseases, targeting a few herpes family viruses. In this study, we analyzed 12 kinds of DNA viruses including 8 species of herpes family viruses in the gastrointestinal mucosa of patients with hematologic malignancies, inflammatory bowel diseases, collagen diseases, or other miscellaneous forms of gastroenteritis using the multiplex virus PCR assay, which we recently developed. The virus PCR assay yielded positive results in 63 of 102 patients; Epstein-Barr virus (EBV) was the most frequently detected, followed by cytomegalovirus (CMV), human herpes virus 6 (HHV-6), HHV-7, parvovirus B19, and herpes simplex virus type 1. The frequencies of viral detection in the 4 diseases were similar involving these 6 viruses. Regarding CMV colitis, the multiplex virus PCR assay was superior to the immunohistopathologic method in detecting CMV. All viruses were more efficiently detected in the mucosa than in the blood in individual patients. These results suggest that CMV, EBV, and HHV-6 were commonly detected in the gastrointestinal mucosa of patients with these 4 diseases, and our multiplex virus PCR assay was useful for the early diagnosis of gastrointestinal virus infection, especially CMV colitis.

## 1. Introduction

Immunocompromised patients are at a risk of viral infection. Viral infections include meningitis/encephalitis caused by herpes simplex virus type 1 and 2 (HSV-1 and HSV-2), human herpes virus type 6 (HHV-6), varicella-zoster virus (VZV), and JC virus (JCV), pneumonia by cytomegalovirus (CMV), herpes zoster/varicella by VZV, herpes simplex by HSV-1 or HSV-2, viral cystitis by adenovirus (ADV) or BK virus (BKV), and colitis by CMV. To make an exact diagnosis of these viral infections, viral genome analysis by polymerase chain reaction (PCR) in individual specimens has been performed. The specimens include cerebrospinal fluid (CSF) for meningitis/encephalitis by HSV-1 [1] and HSV-2 [2],[3], peripheral blood and bronchoalveolar lavage (BAL) fluid [4] for CMV pneumonia [5],[6] and colitis [7],[8], peripheral blood or blister fluid for herpes zoster/varicella [9]–[11] or herpes simplex, and urine for hemorrhagic cystitis [12],[13]. Generally, these specimens can be easily obtained and subjected to PCR assay. While in viral gastroenteritis, the specimen for viral genome examination is obtained through mucosal biopsy during colono- or gastro-endoscopic examination with some difficulties. For this reason, only a small number of studies on gastrointestinal virus infection have been performed [7],[14]–[19], examining CMV [7],[14],[18],[19], Epstein-Barr virus (EBV) [14],[17]–[19] and human herpes virus 6 (HHV-6) infections [14]–[16]. In these studies, CMV, EBV, and HHV-6 were detectable by PCR in the intestinal mucosa in immunocompromised patients, such as those with inflammatory bowel diseases. However, multiplexed intestinal virus analysis involving an increased number of immunodeficient conditions has not been performed.

In recent years, we developed a multiplex virus PCR. The multiplex PCR assay is designed to qualitatively and simultaneously measure the genomic DNA of 12 viruses including CMV and EBV. When a specific PCR signal is obtained, the viral load is determined by a quantitative real-time PCR. It takes only 3 hours to complete both the qualitative multiplex and quantitative real-time PCR procedures [20]. With this assay system, we have analyzed viral infections of the gastrointestinal mucosa in patients after allogeneic HSCT and in those with hematologic malignancies, collagen diseases, inflammatory bowel diseases, and other types of gastroenteritis. The objectives of this study were to investigate the following: (1) The incidence of viral detection and viral species in gastrointestinal mucosa in the above-mentioned diseases; (2) The relationship between CMV detection in the intestinal mucosa and histopathologic diagnosis; (3) Comparison of viral detection between the gastrointestinal mucosa and blood; (4) The possibility of colitis caused by herpes family virus other than CMV. Namely, we conducted the present study to comprehensively elucidate the viral infectious state of gastrointestinal mucosa in these immunodeficient patients or those with miscellaneous gastroenteritis.

## 2. Materials and methods

### 2.1. Patients

All patients were referred to the Laboratory of Cell Therapy in Shinko Hospital by their attending physicians for multiplex virus PCR analysis because of suspected gastrointestinal viral infection (ulcer or mucosal defect) from August 2011 to June 2016. Patients examined in the present study included those who received HSCT, those with hematologic malignancies under chemotherapy, collagen disease under immunosuppressive treatment, inflammatory bowel disease, and miscellaneous gastroenteritis associated with or without melena. The present retrospective analysis was a single institutional clinical study designated the “Multiple virus-analytic study by multiplex virus PCR in immunocompromised patients”, which had been approved by the Ethical Committee of Shinko Hospital.

### 2.2. Gastrointestinal endoscopy and mucosal biopsy

Mucosal biopsy specimens were obtained during endoscopic examination for both histopathologic and viral analyses from individual patients who provided written informed consent for the multiplex PCR examination. Two healthy persons who underwent gastroendoscopy also provided written informed consent for the PCR assay. The size of the mucosal biopsy specimen for the viral PCR was the same as that for histopathologic examination. Colon mucosal viral PCR assay of healthy persons was not performed because the risk of colonal mucosa biopsy in them was controversial among physicians in the Department of Gastroenterology and Hepatology of Shinko Hospital.

### 2.3. Multiplex virus PCR assay

The mucosal specimen was incubated in a mixed solution with 180 μL of Buffer ATL (Qiagen, Tokyo, Japan) and 20 μL of Protease K (Qiagen) at 56 °C until complete lysis of the specimen. Genomic DNA was extracted from the solution using the QIAamp DNA Mini kit (Qiagen), and then subjected to multiplex qualitative PCR and subsequently to quantitative real-time PCR. In each assay, GAPDH was quantified as an internal control, and the viral load is indicated in copy/μg DNA. When the specimen was the peripheral blood, EDTA-2Na-chelated whole blood (200 μL) was obtained from individual patients, and then the plasma was subjected to the PCR assay. The method for both qualitative multiplex PCR and quantitative real-time PCR was previously described in detail [20],[21]. The multiplex PCR was designed to qualitatively detect the genome of 12 DNA viruses including 8 herpes family viruses: CMV, EBV, HHV-6, HHV-7, HHV-8, VZV, BKV, JCV, parvovirus B19 (ParvoB19), HSV-1, HSV-2, and human hepatitis B virus (HBV). When a specific PCR signal was obtained, quantitative real-time PCR was performed to determine the viral load. The lower limits of virus detection using the qualitative multiplex PCR with the plasma specimen were: 1 × 10^3^ copies for CMV, 2.5 × 10^3^ for EBV, 2.5 × 10^3^ for HHV-6, 5 × 10^3^ for HHV-7, 5 × 10^3^ for HHV-8, 1 × 10^3^ for VZV, 1 × 10^3^ for BKV, 5 × 10^3^ for JCV, 1 × 10^3^ for ParvoB19, 2.5 × 10^3^ for HSV-1, 1 × 10^3^ for HSV-2, and 5 × 10^3^ copies/mL plasma for HBV [20],[21].

### 2.4. Pathologic examination of mucosal CMV

Mucosal CMV infection was determined by the presence of CMV-caused inclusion bodies in H-E staining and positive CMV immunostaining with a mouse monoclonal antibody (Roche Diagnostics, Tokyo, Japan)

### 2.5. Statistical analysis

To evaluate differences between rates of patients with positive virus detection, Pearson's Chi-squared test with Yates' continuity correction, Fisher's Exact Test, or the Bonferroni method (for multiple comparison) was employed. To compare mean values, Student's t-test was used.

## 3. Results

### 3.1. Number of patients examined in respective immunodeficient diseases or disorders

A total of 102 patients were examined for possible gastrointestinal viral infection, of whom 81 patients had colon disorders and 21 had upper gastrointestinal disorders. In the 102 patients, the largest number of patients (30 patients) included was those with inflammatory bowel diseases, followed by miscellaneous gastroenteritis with or without melena (26 patients), hematologic malignancies (26 patients) under chemotherapy (15 patients) and post-HSCT (11 patients), and collagen diseases (20 patients).

### 3.2. Incidence of gastrointestinal viral detection

**Figure 1.**
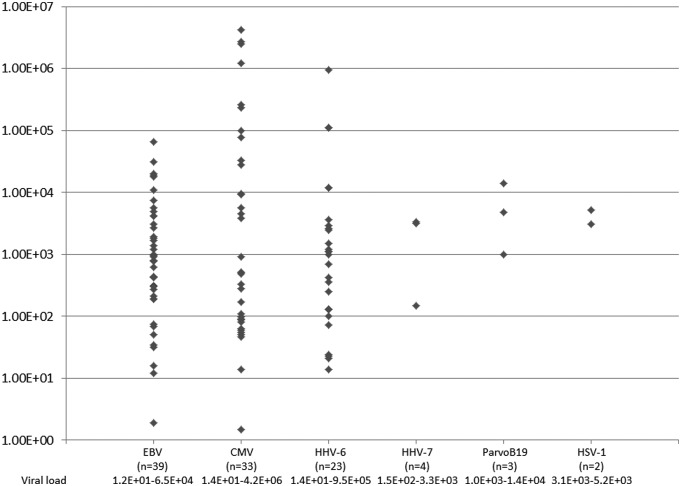
Incidence of gastrointestinal viral detection. A total of 102 patients were examined, of whom 81 and 21 patients had lower and upper endoscopic examinations, respectively. The unit of viral load/copy number is μg DNA. EBV: Epstein-Barr virus; CMV: cytomegalovirus; HHV-6: human herpes virus type 6; ParvoB19: parvovirus B19; HSV-1: herpes simplex virus type 1.

As shown in Figure 1, the gastrointestinal PCR test was performed for a total of 102 patients, and the multiplex qualitative PCR yielded positive results in 62 of the 102 patients. The most frequently detected virus was EBV, in 39 of the 102 patients, followed by CMV (33 patients), HHV-6 (23 patients), HHV-7 (3 patients), ParvoB19 (3 patients), and HSV-1 (2 patients). The virus copy number detected ranged from 1.2 × 10^1^ to 4.2 × 10^6^ copies/μg DNA. In this study, we could detect each virus at levels as low as 1.0 × 10^1^ copies/μg DNA when compared with those in the plasma, although the standard unit was different.

### 3.3. Incidence of virus detection from colon mucosa in immunodeficient diseases or miscellaneous colitis

A total of 81 patients were examined for multiplex virus PCR because of an ulcer or a mucosal defect. Table 1 shows the number of patients in whom one or more viruses were detected from the colon mucosa in the respective diseases/disorders. The number of patients in whom at least one kind of virus was detected was 52 (64.2%). EBV was the most frequently detected, in 31 patients (38.3%), followed by CMV (34.6%), HHV-6 (24.7%), HHV-7 (3.7%), and ParvoB19 (1.2%). The remaining 7 species of viruses screened by the multiplex qualitative virus PCR method were not detected, including HSV-1, which was only detected in the upper gastrointestinal mucosa in a few patients, as described in the following paragraph. The incidence of total virus detection in respective diseases/disorders was not significant. Also, the incidence of the detection of each virus in respective diseases/disorders did not significantly differ among the diseases. In a total of 30 patients (Table 1), more than 2 species were detected in 4 of 8 (50.0%) post-allo-HSCT patients and 6 of 12 (50.0%) in hematologic malignancies under chemotherapy, followed by 7 of 17 (41.2%) in miscellaneous colitis, 11 of 27 (40.7%) in inflammatory bowel diseases, and 2 of 16 (12.5%) in collagen diseases. The incidence of multiple virus detection tended to be low in patients with collagen disease. The combination of EBV and CMV was observed in 15 of 30 multi-detectors (50.0%), and this was significantly higher than that of other combinations (EBV + CMV versus EBV + HHV-6: *p* = 0.041; EBV + CMV versus CMV + HHV-6: *p* = 0.017).

### 3.4. Incidence of virus detection from upper gastrointestinal mucosa in immunodeficient diseases or miscellaneous gastroenteritis

In Table 2, the incidence of virus detection in the upper gastrointestinal mucosa is shown. All 21 patients received endoscopic examination because of epigastric pain or discomfort, nausea, or vomiting. The multiplex virus PCR assay was requested for possible viral infection by respective attending physicians based on mucosal findings (ulcer or mucosal defect) on endoscopic examination. Similar to the incidence of viral detection and virus species in the large intestine, EBV was the most frequently detected, in 8 of 21 patients (38.1%), followed by CMV (23.8%), HHV-6 (14.3%), HSV-1 (9.5%), and ParvoB19 (9.5%). Interestingly, apart from colon analysis, HSV-1 was detected in 2 patients (9.5%), from the mucosa of the esophagus. The pattern of multiple virus detection was similar to that in colon analysis except for a high rate in collagen disease. HSV-1 detection was restricted to those with hematologic malignancies. This unusual virus detection of HSV-1 as well as ParvoB19 and clinical features will be described in the following paragraph.

It was interesting that both HHV-6 and HHV-7 were detected in the gastric mucosa in one of 2 healthy persons, and the HHV-6 load was above 1 × 10^3^ copies/μg DNA. Two years later, similar results were obtained. This person is quite healthy but develops a reddish skin rash each time after febrile common cold-like upper respiratory symptoms, confirming the latency of these 2 viruses.

### 3.5. Detection of HSV-1, ParvoB19 and clinical features

HSV-1 and ParvoB19 were detected in 2 and 3 patients, respectively. In all the 5 patients, the respective viral load exceeded 1 × 10^3^ copies/μg DNA (Figure 1). HSV-1 detection was restricted to 2 patients with hematologic diseases (post-allogeneic HSCT for acute myeloid leukemia and severe aplastic anemia), and also restricted to the esophagus as the site of detection. While ParvoB19 detection was observed in a variety of diseases (myeloid leukemia terminated from myelodysplastic syndrome, enteritis without underlying disease, and rheumatoid arthritis), and the site of virus detection was the terminal ileum, duodenum, and stomach, respectively (Tables 1 and 2). Interestingly, in these 5 patients, there was no skin or mucosal blister caused by HSV-1, skin erythema, or pure red cell aplasia due to ParvoB19, suggesting local infection or colonization by these 2 viruses. Re-examination was not performed because of improved symptoms with supportive care (3 patients) or the deterioration of underlying diseases (2 patients).

**Table 1.** Incidence of virus detection from colon mucosa in immunodeficient diseases or miscellaneous colitis.

Disease/Disorder	No. of patients	Detection/patients	Cumulative No. of patients with positive viral detection					Multiple virus detection	
			EBV	CMV	HHV-6	HHV-7	ParvoB19	No. of patients	Total (%)
Hematologic malignancy: Post-allo-HSCT	8	6/8 (75.0%)	2	3	4	1	0	EBV + CMV: 1	4/8 (50.0%)
								CMV + HHV-6: 1	
								EBV + HHV-6: 1	
								CMV + HHV-7 + HHV-6: 1	
Hematologic malignancy: Post-auto-HSCT	1	0/1 (0%)	0	0	0	0	0		
Hematologic malignancy: under chemotherapy	12	9/12 (75.0%)	7	7	2	0	1	EBV + CMV: 4	6/12 (50.0%)
								EBV + HHV-6 + ParvoB19: 1	
								CMV + HHV-6: 1	
Collagen disease	16	10/16 (62.5%)	5	4	4	0	0	CMV + HHV-6: 1	2/16 (12.5%)
								EBV + CMV + HHV-6: 1	
Inflammatory bowel disease	27	17/27 (63.0%)	11	9	6	1	0	EBV + CMV: 6	11/27 (40.7%)
								EBV + CMV + HHV-6: 2	
								EBV + HHV-6: 2	
								CMV + HHV-6: 1	
Miscellaneous colitis	17	10/17 (58.8%)	6	5	4	1	0	EBV + CMV: 4	7/17 (41.2%)
								EBV + HHV-6: 2	
								EBV + CMV + HHV-6: 1	
Total	81	52/81 (64.2%)	31 (38.3%)	28 (34.6%)	20 (24.7%)	3 (3.7%)	1 (1.2%)	EBV + CMV: 15/30 (50.0%)	30/81 (37.0%)
								EBV + HHV-6: 5/30 (16.7%)	
								CMV + HHV-6: 4/30 (13.3%)	

Allo-HSCT: allogeneic hematopoietic stem cell transplantation, auto: autologous.

**Table 2.** Incidence of virus detection from upper gastrointestinal mucosa in immunodeficient diseases or miscellaneous gastroenteritis.

Disease/Disorder	No. of patients	Detection/patients	Cumulative No. of patients with positive viral detection						Multiple virus detection	
			EBV	CMV	HHV-6	HSV-1	ParvoB19	HHV-7	No. of patients	Total (%)
Hematologic malignancy: Post-allo-HSCT	2	2/2	1 (e:1)	1 (d:1)	1 (d:1)	1 (e:1)	0	0	HSV-1 + EBV: 1 (e) CMV + HHV-6: 1 (d)	2/2 (100%)
Hematologic malignancy: under chemotherapy	3	2/3	2 (e:2)	0	1 (e:1)	1 (e:1)	0	0	EBV + HHV-6: 1 (e) HSV-1 + EBV: 1 (e)	2/3 (66.7%)
Collagen disease	4	4/4	4 (e:1, s:1, d:2)	3 (e:1, d:2)	1 (e:1)	0	1 (s:1)	0	EBV + CMV: 2 (d) EBV + CMV + HHV-6: 1 (e) EBV + ParvoB19: 1 (s)	4/4 (100%)
Inflammatory bowel disease	3	0/3	0	0	0	0	0	0		
Miscellaneous gastroenteritis	9	2/9	1 (s:1)	1 (s:1)	0	0	1 (d:1)	0		
Healthy person	2	1/2	0	0	1 (s:1)	0	0	1 (s:1)	HHV-6 + HHV-7: 1 (s)	1/2 (50%)
Total	21	10/21 (47.6%)	8 (38.1%)	5 (23.8%)	3 (14.3%)	2 (9.5%)	2 (9.5%)		EBV + CMV: 2/8 (25.0%); EBV + HHV-6: 1/8 (12.5%)	8/21 (38.1%)

e: esophagus, s: stomach, d: duodenum.

### 3.6. Relationship of mucosal virus detection between upper gastrointestinal and lower intestinal tracts

Three patients were examined with multiplex virus PCR for both the upper gastrointestinal tract and colon with a close interval. No specific treatment was performed during the interval in these patients. In one patient with Behcet's disease, EBV (7.9 × 10^2^ copies/μg DNA) and EBV (1.2 × 10^1^ copies) plus CMV (9.3 × 10^3^ copies) were detected in colon mucosa and gastric mucosa, respectively. Another patient with Behcet's disease showed negative virus detection in both lower and upper gastrointestinal tracts. These 2 patients showed a similar pattern of detection in terms of positive results in the former and negative results in the latter in both the upper and lower tracts. While in a patient with Crohn's disease, EBV (3.4 × 10^1^ copies) and CMV (3.3 × 10^2^ copies) were only detected in the colon mucosa.

### 3.7. CMV detection in the colon mucosa (Table 3) and antiviral treatment

CMV was detected in the colon mucosa in 29 patients, as shown in Table 3. As described in the previous paragraph, the incidence of CMV detection in respective diseases or disorders did not significantly differ. The CMV copy number ranged from 1.4 × 10^1^ to 4.2 × 10^6^ copies/μg DNA. In this analysis, we tentatively posed the CMV copy number of more or less than 1 × 10^3^ copies/μg DNA to determine CMV disease or latency. The incidence of melena and fever did not significantly differ between patients with more or less than 1 × 10^3^ CMV copies. The levels of C-reactive protein (CRP) were also not significantly different between the 2 groups. Immunohistopathologic diagnosis of CMV colitis was made in 6 of 13 patients (46.2%) in whom more than 1 × 10^3^ CMV copies were detected, while the diagnosis was made in only 3 of 16 patients (18.8%) with a load of less than 1 × 10^3^ CMV, without a significant difference (data not shown).

Thirteen of 29 patients in whom CMV was detected by multiplex PCR assay were treated with ganciclovir based on histopathologic diagnosis or positive PCR result with improved abdominal distress. However, remaining 16 patients including 5 with more than 1 × 10^3^ CMV copies, also recovered from abdominal distress with supportive treatments suggesting that more or less 1 × 10^3^ CMV copies could not be a turning point between CMV disease and latency.

### 3.8. Comparison of incidence of virus detection between the intestinal mucosa and blood

In some patients, virus PCR tests were simultaneously performed with intestinal mucosa and blood specimens. Therefore, we compared the incidence of virus detection between the intestine and blood. EBV was not detected in the blood in 4 of 10 patients in whom EBV was detected in the colon mucosa. CMV was not detected in the blood in 2 of 10 patients with positive mucosal CMV. As for HHV-6, this virus was not detected in the blood in 8 patients with detectable mucosal HHV-6. Conversely, only a small number showed blood viral detection among mucosal virus-negative patients (data not shown), indicating the higher detection of these viruses in the colon mucosa than in the blood.

### 3.9. Patients with miscellaneous colitis without CMV detection (Table 4)

CMV colitis is an established clinical entity; however, whether gastroenteritis is caused by other herpes-family viruses is unknown. As shown in Table 4, in 5 patients but not Patient D, who had abdominal distress with an ulcer or a mucosal defect on endoscopy and with a negative fecal microorganism culture, HHV-6 or EBV was detected in the colon mucosa with a relatively high viral load, at mostly above 1.0 × 10^3^ copies/μg DNA. In Patient C, both HHV-6 and EBV were detected. These patients, however, did not have a skin rash or atypical lymphocytes in the peripheral blood, suggesting local viral infection, and all 4 patients recovered with fluid therapy. Patient D with an excised MALT lymphoma did not have abdominal distress, but underwent colonofiberscopy and mucosal biopsy for evaluation after the lymphoma excision. Although the biopsied mucosa looked normal, HHV-7 was detected with a viral load of 3.2 × 10^3^ copies/μg DNA, suggesting the mucosal latency of this virus.

**Table 3.** CMV detection in the colon mucosa and clinical features.

Disease/Disorder	No. of patients	Median CMVcopy number (range)	Melena	Fever	Median CRP (mg/dL) (range)	CMV histopathologic detection		Antiviral agents	Outcome
						HE	Immunostaining		
Post-allo-HSCT	4	1.7E+04 (5.0E+01∼2.7E+06)	3/4	1/4	4.8 (0.6∼8.4)	2/4	3/4	2/4	Improve (4/4)
Hematologic malignancy under chemo	6	1.7E+05 (4.7E+01∼2.5E+06)	4/6	2/6	8.1 (0.9∼11.8)	3/6	4/6	3/6	Improve (6/6)
Collagen disease	4	7.6E+03 (6.3E+01∼2.3E+05)	2/4	1/4	6.5 (0.6∼8.7)	0/3	2/3	2/4	Improve (4/4)
Inflammatory bowel disease	9	2.8E+03 (5.5E+01∼2.8E+04)	5/9	2/9	7.6 (0.5∼18.1)	0/9	0/9	5/9	Improve (9/9)
Gastroenteritis	6	2.1E+03 (1.4E+01∼4.2E+06)	3/6	2/6	8.3 (0.2∼39.7)	0/6	0/6	1/6	Improve (6/6)

Units of CMV copy number and CRP are µg DNA and mg/dL, respectively. Abdominal distress of all patients treated with ganciclovir was improved. Remaining patient also recovered from their abdominal distress with supportive treatments.

**Table 4.** Patients with miscellaneous colitis without CMV detection and their mucosal viral status and clinical picture.

Patient	Final diagnosis	Virus detection (co-detection)	Viral road	Symptoms	Endoscopy & histology	CRP	Atypical lym	Treatment	Outcomes
A	Ischemic colitis	HHV-6	1.1E+03	Fever, abdominal pain, vomiting	Mucosal defect Nonspecific inflammation	4.9	0%	Supportive care	Recovery
B	Mesenteric phlebosclerosis	HHV-6	2.6E+03	Fever, abdominal pain, diarrhea	Mucosal defect Nonspecific inflammation	12.0	Unknown	Fluid, antibiotics	Recovery
C	Sigmoid diverticulitis	HHV-6 EBV	1.5E+03 4.2E+03	Fever, abdominal pain	Mucosal defect Colitis	11.2	0.5%	Antibiotics	Recovery
D	MALT lymphoma post-excision	HHV-7	3.2E+03	None	Normal mucosa	0.03	0%	None	No symptom
E	Colon ulcer	EBV	1.9E+02	Abdominal pain, vomiting	Ulcer Colitis	26.5	0%	Fluid, antibiotics	Recovery

## 4. Conclusions

In the present study, we examined gastrointestinal mucosa for possible target for viral infection. Thus, we have detected viral DNA with using the multiplex virus PCR assay in patients with an immunodeficient disease or status. There have been a few studies regarding gastrointestinal EBV, CMV, or HHV-6 infection using a quantitative PCR method that can assay a single virus. In the present study, we simultaneously assayed 12 kinds of viruses including these 3 viruses in the gastrointestinal mucosa involving a larger number of diseases or statuses than examined previously. Therefore, this is the first report of a study on many kinds of gastrointestinal virus infections in patients with various abnormal immune statuses. The detected viruses in the present study in both lower and upper gastrointestinal tracts were EBV, CMV, HHV-6, HHV-7, HSV-1, and ParvoB19, suggesting that these 6 but not the remaining 6 kinds of virus are involved in immunodeficiency-related gastroenteritis.

In the large intestine, the most frequently detected virus was EBV, followed by CMV, HHV-6, HHV-7, HSV-1, and ParvoB19 (Table 1). The detection of HHV-7, HSV-1, and ParvoB19 in the gastrointestinal mucosa has not been reported. The incidence of virus detection was highest (75%) in patients with post-allo-HSCT, who were considered to show the most immunodeficient status; however, the incidence was not significantly different from that in patients with other diseases or statuses, presumably due to the small number of patients examined in the present study. From another point of view, however, virus infection or detection may be common not only in post-allo-HSCT but also in other abnormal immune statuses, even in patients with gastroenteritis without underlying disease (Table 1). Dual or multiple virus detection was frequently observed, with the highest rate in patients post-allo-HSCT (50%), although the incidence was also not significantly different, presumably for the same reason. However, the combination of EBV and CMV was significantly higher among other combinations of virus detection. This might be due to the relatively high incidence of infection or latency of EBV [17] and CMV [22] in the gastrointestinal mucosa in patients with an abnormal immune status.

It should be noted that the incidence of virus detection was high in patients with miscellaneous colitis (58.8%), showing a similar pattern of detection to other diseases/statuses in terms of the virus species detected and frequent combination of EBV and CMV. The majority of these patients did not have underlying disease and their abdominal distress including melena was transient, being improved with conservative treatment. Thus, in a future study, follow-up of virus PCR examination will be needed to determine the role of these viruses in this type of colitis.

As for exceptional virus detection, HSV-1 could chronically affect the GI tract in immunocompromised hosts without severe symptoms, as observed in patients in the present study [23]. ParvoB12 detection in the GI tract has not been reported. Regarding the cellular origin of the ParvoB12 detection in the present study, endothelial cells in the GI tract may be likely because they express P antigen, which is necessary for the entry of ParvoB19 into cells [24]. Indeed, ParvoB19 detection was reported in endothelial cells of the heart and liver [25], and the placenta [26]. In addition, it was ruled out that ParvoB19 detectors in the present study were the carrier of the virus because the prevalence of DNA detection in blood donors is low, being 0.003 to 0.6% [27].

We simultaneously performed the multiplex virus PCR assay in both upper and lower gastrointestinal tracts. The virus detection showed roughly similar patterns at both sites. Therefore, it is of marked interest that an upper GI tract virus examination could be employed as a substitute for colonofiberscopy because a high CMV detection rate in upper GI tract biopsies from heart transplant patients was reported [28].

In the diagnosis of CMV, there was a discrepancy between the results of our PCR assay and immunohistopathologic diagnosis. Generally, multiple antibodies are necessary to perform a precise immunohistopathologic procedure [29]; therefore, it may be reasonable that the PCR assay yielded more positive results. In addition, a higher sensitivity of PCR compared with that of immunohistochemistry in detecting CMV in upper GI and liver transplant biopsies was reported [28],[30]. In the present study, attending physicians for respective patients decided on ganciclovir treatment based on the patients' symptoms, especially melena, and CMV detection by PCR. The indication of ganciclovir treatment should be determined in the future in relation to CMV copy number.

We analyzed the possibility of colitis caused by a herpes family virus other than CMV. In these patients with miscellaneous colitis (Table 4), an ulcer or a mucosal defect was endoscopically observed with a negative fecal culture of microorganisms, suggesting mucosal viral infection. Therefore, local and mucosal viral infection or re-activation of HHV-6 and EBV should be taken into consideration, considering that 22 of 64 patients (34.4%) with underlying diseases showed a negative PCR result in the colon mucosa. Indeed, HHV-6 colitis [15] or severe colitis associated with EBV and CMV reactivation [8] was reported.

The limitations of this study include: retrospective analysis, too small patient population to draw a definite conclusion, unclear indication of gastrointestinal virus PCR, vague indication for ganciclovir treatment in patients with positive colonal CMV, and unclear criteria for virus infection or latency. In the future, the significance of viral detection in the gastrointestinal mucosa and appropriate therapeutic indications for antiviral treatment should be determined with a prospective study.
